# Supplementary material for: High Throughput Identification of the Potential Antioxidant Peptides in Ophiocordyceps sinensis
Source: Molecules. 2022 Jan 10;27(2):438. doi: 10.3390/molecules27020438 (PMC8780859; doi:10.3390/molecules27020438)
Supplement: Supplementary file 1 [file molecules-27-00438-s001.zip › Table S9. Primers used for quantitative RT-PCR.pdf]

Table S9

Gene ID	Primers
MSTRG.1154.1	Forward primer 5’-TCGTCTTCAAAGCCAAGAGGT-3’
	Reverse primer 5’-AGCTCACTGTCCGATAAACAG-3‘
MSTRG.7028	Forward primer 5‘-CGCTTTTGTTTGGTCGTCT-3‘
	Reverse primer 5’-GCAGAAATTGCCCTCGTTC-3’
MSTRG.5117	Forward primer 5'-CTTTCCGGATCTCCAGGTCG-3'
	Reverse primer 5'-TGATCCGGCAACGAGAGAAG-3'
MSTRG.10287	Forward primer 5’-CGAGCATTGCGTCCTCATTC -3’
	Reverse primer 5’-CAGAGTTGATGGGTCGGCAT -3’
MSTRG.9713	Forward primer 5’-ATTGGCAAATCCGTTGCAC -3’
	Reverse primer 5’-TAGTCGGGCTTTGGTTGGAG -3’
MSTRG.9912	Forward primer 5'-TCTCCTCCTTCCTTGTTTGC-3'
	Reverse primer 5'-AATGGCAATGGCGCAGTAG-3'
MSTRG.10795	Forward primer 5'-CCTCACCACTACCTCTTGAC-3'
	Reverse primer 5'-ATGTCCCTACCGAGATCCGAG-3'
MSTRG.5870	Forward primer 5'-ACAGAGCAAGTCGAGCAACG-3'
	Reverse primer 5'-CTTGCTTGCAGGTATCCGAC-3'
MSTRG.1158.1	Forward prime 5'-GAATATTGGCGCTTCCGCTG-3‘
	Reverse primer 5’-CTCGGAGGCGTTCTTGATGT-3‘
